# Vibrational communication between a myrmecophilous butterfly *Spindasis lohita* (Lepidoptera: Lycaenidae) and its host ant *Crematogaster rogenhoferi* (Hymenoptera: Formicidae)

**DOI:** 10.1038/s41598-019-54966-6

**Published:** 2019-12-06

**Authors:** Yueh-Hsien Lin, Yi-Chang Liao, Chin-Cheng Scotty Yang, Johan Billen, Man-Miao Yang, Yu-Feng Hsu

**Affiliations:** 1 College of Life Science, National Taiwan Normal University, 88, Sec. 4, Tingzhou Rd., Taipei, 116 Taiwan; 2 Department of Entomology, National Chung Hsing University, 145 Xinda Rd., Taichung, 402 Taiwan; 3 Research Institute for Sustainable Humanosphere, Kyoto University, Gokasho, Uji, Kyoto, 611-0011 Japan; 4 KU Leuven, Zoological Institute, Naamsestraat 59, box 2466, B-3000 Leuven, Belgium

**Keywords:** Entomology, Animal behaviour

## Abstract

Ants are a dominant insect group in terrestrial ecosystems and many myrmecophilous species evolve to associate with ants to gain benefits. One iconic example is myrmecophilous butterflies that often produce ant-mimicking vibrational calls to modulate ant behaviors. Despite its popularity, empirical exploration of how butterflies utilize vibrational signals to communicate with ants is scarce. In this study, we reported that the myrmecophilous butterfly *Spindasis lohita* produce three types of larval calls and one type of pupal call, while its tending ant, *Crematogaster rogenhoferi* emit a single type of call. The results of discriminant analysis revealed that calls of the two species are quantitatively similar in their signal attributes; the potential role of butterfly calls are further confirmed by the playback experiments in which certain ant behaviors including antennation, aggregation, and guarding were induced when one of the butterfly calls was played to *C*. *rogenhoferi* workers. The findings in the current study represent the very first evidence on vibrational communication between *Spindasis* and *Crematogaster* and also imply that *S*. *lohita* may have been benefited from ant attendance due to the ability to produce similar calls of the ant *C*. *rogenhoferi*.

## Introduction

Ants are thought to be one of the most dominant insect groups in terrestrial ecosystems due to their sophisticated caste and complex communication systems^1^. Animals that evolve behaviors to associate with ants to gain benefits are termed myrmecophiles. To evolve myrmecophily, an organism by necessity acquires the ability to decode the ants’ communication^2^. In other words, myrmecophiles have attained the ability to speak the ants’ language either mechanically or chemically^1^. Many arthropods are known to be myrmecophiles, and can be generally divided in five groups based on their behaviors^3^: (1) synechthrans are predators of ants or their broods, using high speed or repellent secretions to avoid the ants’ attack; (2) synoeketes are mostly scavengers and predators who often tend to be ignored by their hosts due to neutral odor or no odor; (3) symphiles, also referred to as true guests, are accepted to some extent by their host and usually consume their host or brood for living; (4) ectoparasites and endoparasites whose life cycles rely on their host through parasitism. They live inside the body surface of the host and lick up their oily secretions or bite through their exoskeleton to live; and (5) trophobionts, in which phytophagous homopterans, heteropterans and lycaenid caterpillars (such as the focal system in the current study) are included. They supply their host with honeydew and nutritive glandular secretions in exchange for protection.

Caterpillars are usually rather slow-moving insects with a soft cuticle and thus are a prototype of ant prey. Ants exert a substantial selective pressure on lepidopteran larvae^4,5^. Myrmecophily, however, is widespread within Lepidoptera, particularly for the immature stages of Riodinidae and Lycaenidae^6,7^, which are often globally referred to as “lycaenoids”^8^. More than 50% of lycaenid butterfly species interact with ants during part of their life cycle^7,9,10^, with their relationships varying from facultative to strictly obligate mutualistic or parasitic to the associated ants. Most of these lycaenid larvae have developed myrmecophilous organs to attract ants to tend either by providing food resources or mimicking chemical signals of ants^11–13^. Consequently, lycaenid larvae are generally believed to gain life history advantages (e.g., reduction of risk of predation or parasitism) from such protection provided by tending ants^11,14–16^. In some extreme cases, caterpillars of certain species are more vulnerable to predation if they are not tended by the ants^11,17^, indicating that efficiency of caterpillar in attracting ants plays as a key fitness determinant for myrmecophilous caterpillars. One of the most well-studied systems in which bidirectional communication channels are investigated concerns parasitic *Maculinea* caterpillars and their *Myrmica* host ants^18–20^.

As commonly seen in other insect herbivores, myrmecophilous caterpillars may have developed vibrational behaviors to attract ants. One example is that larvae of a riodinid *Thisbe irenea* are capable of using special vibrational papillae to produce vibrational calls^21^, and that caterpillars of this species generally attract significantly more ants than “mute” caterpillars, suggesting the vibrational papillae may function as an ant-attraction organ^21^. The most straightforward evidence was derived from Travassos and Pierce^22^ showing that both larvae and pupae of the common imperial blue butterfly *Jalmenus evagoras* regulate the number of attendant ants, *Iridomyrmex anceps* using substrate-borne vibration. Moreover, larvae and pupae of another parasitic butterfly *Maculinea rebeli* were reported to produce a signal that mimics the queen of their host ant, *Myrmica schencki* to attain high social status in the colony^18^. These findings, along with other recent ones^23–25^, suggest that utilization of vibrational calls may be a common phenomenon between myrmecophilous butterflies and ants.

Many lycaenid butterflies in Taiwan are associated with ants, and their caterpillars were usually found to use myrmecophilous organs such as dorsal nectary organs (DNOs) to maintain the level of attendant ants^7^. Nevertheless, Wang^26^ reported that no secretion organ was found on the pupa in one of these Taiwanese lycaenid butterfly species, namely the long-banded silverline, *Spindasis lohita*.

The butterfly *S*. *lohita* is widely distributed in the montane areas of Taiwan and females only lay eggs on host plants with the presence of nest of the tending ant *C*. *rogenhoferi*^26^. The caterpillars can be found in the shelter made of leaves near the main nest of *C*. *rogenhoferi* or within the shallow layer of the nest. The caterpillars are usually tended by the ants in the wild and the mortality is significantly higher if attending ants are excluded^26^. These findings suggest that the butterfly *S*. *lohita* and the tending ant *C*. *rogenhoferi* have developed a one-to-one relationship. Moreover, the pupae were tended by ants at a similar level as larvae, leading us to hypothesize that pupae (and possibly larvae) of this butterfly may produce specific vibrational calls to attract their tending ant, the acrobat ant *C*. *rogenhoferi*. Hence, in this study we attempted (1) to show that the larvae and pupae of *S*. *lohita* can make specific vibrational calls; (2) to demonstrate the vibrational calls emitted by *S*. *lohita* are capable of attracting ants; (3) to test whether the ant *C*. *rogenhoferi* can make communication calls; and (4) to characterize potential signal-producing structures of the ant *C*. *rogenhoferi*. For (2) and (3), we used playback experiments to test if the calls can trigger benevolent behaviors of the ant. Playback experiments are essential for testing hypotheses regarding receiver responses to signals, which include how signals function, what forms of selection that receivers might impose on signals, and how receivers are influenced by signal changes^27^.

## Results

### Vibrational behavior and signals

**Figure 1:**
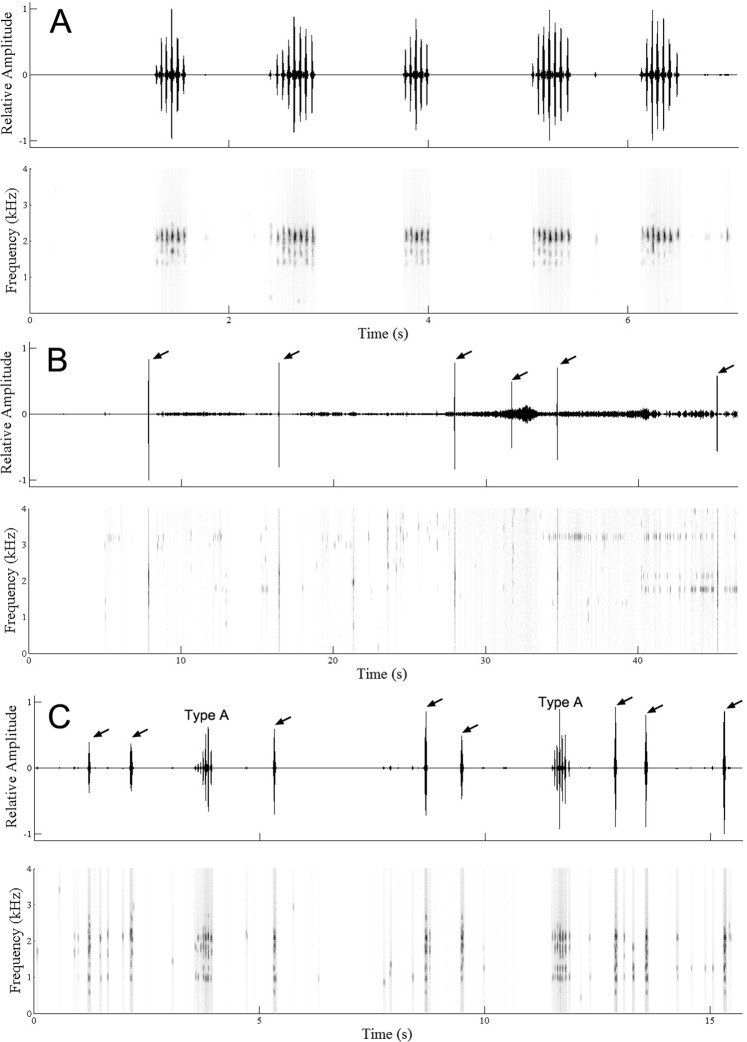
The three types of calls of larvae of *Spindasis lohita*. (**A**) Five type A calls are shown; (**B**) Type B calls, as indicated by arrows; (**C**) Type C calls, as indicated by arrows. Top: oscillogram; bottom: spectrogram.

**Figure 2:**
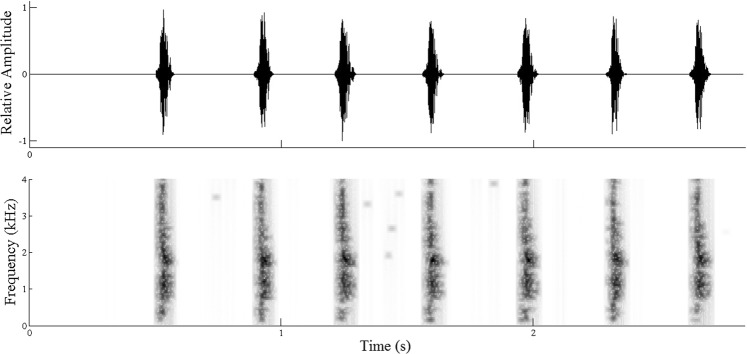
The oscillogram (top) and spectrogram (bottom) of pupal calls of *Spindasis lohita*. A total of seven individual calls are shown.

The larvae of *S. lohita* produced three types of calls (Type A, B, and C) and pupae produced one type of call (Table 1, Figs. 1 and 2, also see Supplementary Audio S1–4). The larvae produced calls during moving, resting and even eating. The larvae emitted Type A calls constantly, and sometimes produced Type B calls. The type A call (*N* = 6, Σ = 82 pulses) consisted of a long pulse train, which resembles to rapid drumming, while Type B calls consisted of a single pulse (*N* = 4, Σ = 45 pulses) that resembles to a grunt. Type C calls also consisted of a single pulse (*N* = 6, Σ = 61 pulses) and occurred randomly. Type C calls were recorded between Type A and B calls intermittently. Despite no obvious abdominal segment contraction, pupae (*N* = 4, Σ = 88 pulses) emitted calls (Fig. 2) immediately once they were properly settled for the recording. Pupae calls resemble to finger snaps and occurred constantly.

**Table 1:** Call characteristics of *Spindasis lohita* and *Crematogaster rogenhoferi* (mean ± SD).

Call	Dominant Frequency (kHz)	Duration (s)	Pulse period (s)
Larva Type A	2.052 ± 0.135a	0.33 ± 0.10a	1.82 ± 0.92b
Larva Type B	1.723 ± 0.318ab	0.02 ± 0.02c	1.86 ± 3.96a
Larva Type C	0.774 ± 0.347c	0.06 ± 0.01b	—
Pupa	1.692 ± 0.291b	0.04 ± 0.03c	0.63 ± 0.56a
Worker ant	1.904 ± 0.274ab	0.11 ± 0.03 (part1)b	3.40 ± 5.14b
		0.35 ± 0.18 (part1 + 2)a	

Characters were compared using the Kruskal-Wallis test. Means followed by different lowercase letters in the same column indicate significant differences among different calls.

**Figure 3:**
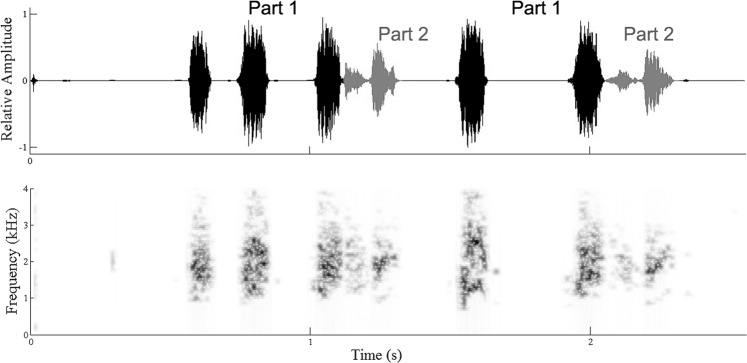
The oscillogram (top) and spectrogram (bottom) of calls of *Crematogaster rogenhoferi*.

The ant workers produced calls by lifting their gaster and rubbing it against the postpetiole. During the recording sessions, the ants only made calls when being disturbed (tapping on the leaves) or when discovering food. Since tapping the leaves would incur extra noise and lead to stylus instability, mealworms were instead provided as food resource on the leaves to stimulate ant calls. Only a single type of call was observed in the workers (*N* = 3, Σ = 62 pulses), and consisted of two parts: part 1 occurred constantly and is sometimes followed by part 2 (Fig. 3, see Supplementary Audio S5). Both parts resemble to rubbing plastic material by human hands (Table 1, Fig. 3). No significant difference was found in dominant frequency (Mann-Whitney *U* test, *U* = 467.5, *P = *0.949) and pulse period (Mann-Whitney *U* test, *U* = 412.5, *P = *0.398) between part 1 and part 1 + 2 of ant calls. The duration of part 1 + 2 was significantly longer than that of part 1 (Mann-Whitney *U* test, *U* = 6, *P < *0.001). It is worth noting that we failed to record any call signals emitted by queens despite the same recording effort made to queens and the presence of a stridulation apparatus also in queens (see further).

Type A and Type C calls possessed the highest and lowest dominant frequency, respectively, among the five recorded call types (Table 1, Kruskal-Wallis test, H = 194.1, *P* < 0.001). For the duration of calls, Type A calls and ant calls (part 1 + 2) were the longest, while Type B calls and pupal calls were the shortest (Table 1, Kruskal-Wallis test, H = 286.5, *P* < 0.001). The pulse period of Type A and the ant calls were significantly different from that of both Type B calls and pupal calls (Table 1, Kruskal-Wallis test, H = 102.2, *P* < 0.001).

**Figure 4:**
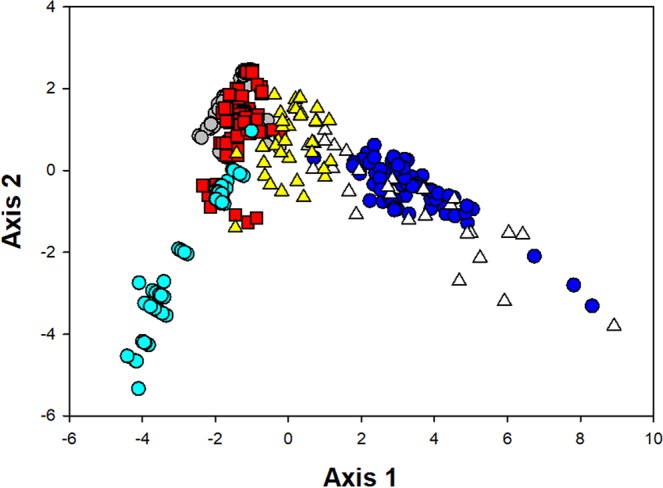
Scatterplot of the first two axes of the discriminant analysis on the calls of *Spindasis lohita* and *Crematogaster rogenhoferi*. Each data point represents a single signal. Blue circle: Type A call of *S*. *lohita*; grey circle: Type B call of *S*. *lohita*; aqua blue circle: Type C call of *S*. *lohita*; red square: pupal call of *S*. *lohita*; white triangle: call (part 1 + part 2) of the ant *C*. *rogenhoferi*; yellow triangle: call (part 1) of ant *C*. *rogenhoferi*.

The result of discriminant analysis on the three call characteristics (dominant frequency, pulse duration and pulse period) of *S*. *lohita* and *C*. *rogenhoferi* is shown in Fig. 4. The call (part 1 + 2) of *C*. *rogenhoferi* overlapped with the Type A call of *S*. *lohita*, whereas Type B calls and pupal calls of *S*. *lohita* were grouped together with the call (part 1 alone) of *C*. *rogenhoferi*. Correct grouping in the discriminant analysis of these signals was 68.32%. All these data suggest that the vibrational characteristics of the lycaenid calls and the ant calls are similar.

### Playback experiments

**Figure 5:**
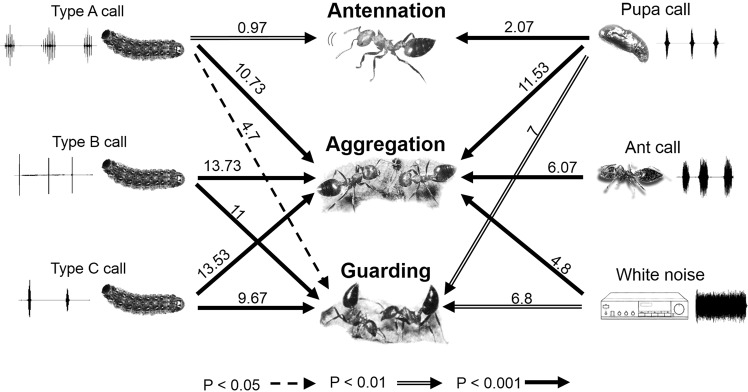
Association between three behaviors of the ant *Crematogaster rogenhoferi* and various playback treatments (i.e., different calls of larvae and pupae of *Spindasis lohita*, ant calls and white noise) that were analyzed using multiple linear regression. Numbers above arrows represent regression coefficients. (The ant, caterpillar, and pupa pictures have been produced by photo courtesy of Chun-Kai Wang).

Among all the experiments, attack behavior was not observed. The occurrence of ant antennation was positively correlated with playback of larval Type A calls and pupal calls (Fig. 5, see Supplementary Table S1). The positive correlation of ant aggregation occurred with all six playback signals, especially for the butterfly’s calls (three larval calls and a pupal call) (Fig. 5, see Supplementary Table S1). The occurrence of ant guarding was positively correlated with all playback signals except ant signals (Fig. 5, see Supplementary Table S1). Type A calls and pupal calls of *S*. *lohita* positively induced three ant behaviors (aggregation, antennation and guarding behavior), while Type B calls and Type C calls of *S*. *lohita* were positively correlated with ant aggregation and guarding behavior. Ant calls had only one positive correlation with ant aggregation.

### Stridulatory structure of the ants

**Figure 6:**
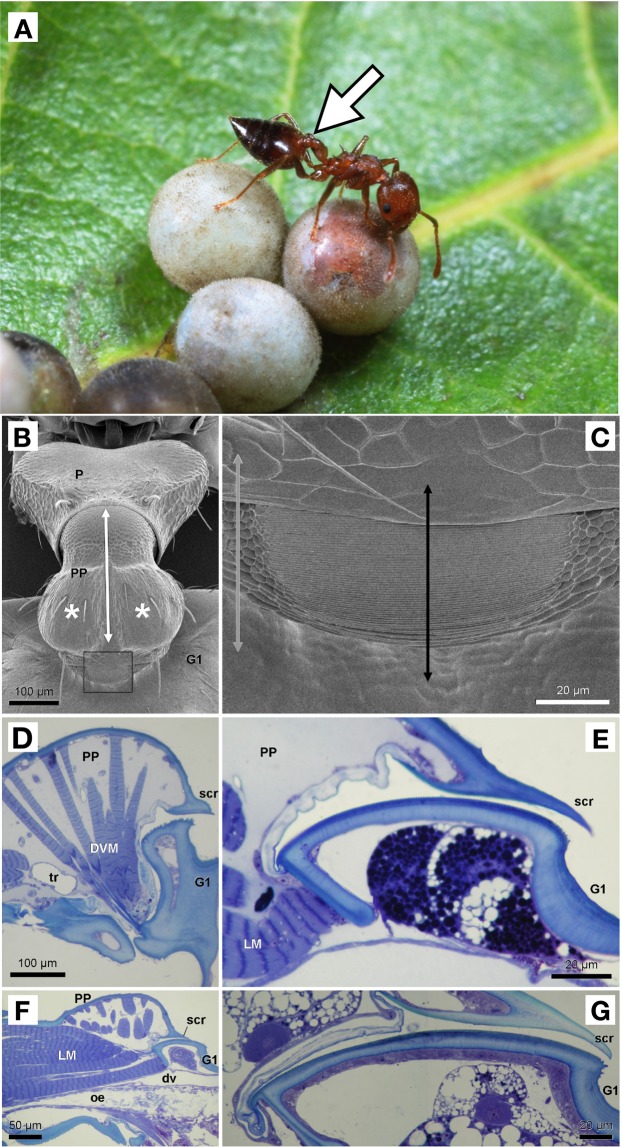
Scanning micrograph of stridulation apparatus of the ant *Crematogaster rogenhoferi*. (**A**) An individual worker of *C*. *rogenhoferi* with a white arrow indicating where the stridulation apparatus is located (postpetiole as shown in 6B) (photo courtesy of Yi-Hui Wu); (**B**) Stridulation apparatus of worker. White asterisks indicate insertion site of paired dorsoventral muscles, white double arrow indicates location of unpaired longitudinal muscle; (**C**) Detail view of framed part in 6B. Grey double arrow indicates plane of sectioning aside of stridulation file as shown in 6D, black double arrow indicates plan of sectioning through stridulation file as shown in 6E-G; (**D**) Longitudinal section through postpetiole of worker, showing dorsoventral muscles to pull anteroventral part of gaster forward (and stridulation file backward). Note irregular appearance of stridulation file region as section corresponds with grey double arrow in 6B; (**E**) Detail of stridulation file of worker; (**F**) Longitudinal section through postpetiole of worker, showing longitudinal muscle to pull anterodorsal part of gaster (and stridulation file) forward; (**G**) Detail of stridulation file of queen. dv: dorsal vessel; DVM: dorsoventral muscle, G1: first gastral segment; LM: longitudinal muscle; oe: oesophagus; P: petiole; PP: postpetiole; scr: scraper; tr: trachea.

Scanning microscopy showed the existence of a prominent stridulation apparatus on the mesodorsal part of the first gastral segment on worker and queen of *C*. *rogenhoferi* (Fig. 6). This consists of a stridulation file with parallel transverse ridges on the anterior part of the first gastral tergite and a scraper that is formed by the posterior and slightly downward-bent edge of the postpetiole (Fig. 6B,C). The stridulation signal is produced by moving the scraper over the file, which is achieved by the action of two antagonistic muscles. Both in the worker and the queen, these consist of two paired laterally located dorsoventral muscles and an unpaired centrally located longitudinal muscle (Fig. 6D,F).

The full size of the stridulation file, however, is usually not visible on scanning micrographs as its anterior portion is hidden underneath the postpetiole. We therefore performed histological examination to view the entire stridulation file. This revealed that queens had a larger stridulation file with more ridges and wider spacing of the ridges than workers (Fig. 6E,G; Table 2).

**Table 2:** Characteristics of the stridulatory file of two queens and four workers (DQ: dealate queen, AQ: alate queen, W1–4: workers 1 to 4) measured from histological semithin sections.

	Length of stridulation file (µm)	Number of ridges	Distance between ridges (µm)
DQ	137	146	0.94
AQ	167	194	0.86
W1	83	116	0.72
W2	106	134	0.79
W3	90	129	0.70
W4	60	90	0.67

## Discussion

We found that the lycaenid *S*. *lohita* produce a total of four types of call including three larval calls and one pupal call, while the ant *C*. *rogenhoferi* produces one type of call (Table 1; Figs. 1–3). With playback experiments and behavioral assays, this study demonstrated the vibrational communication between *Spindasis* and *Crematogaster* for the first time (Fig. 5). Furthermore, the discriminate analysis also revealed the similarity between the calls of *S*. *lohita* and *C*. *rogenhoferi* in their signal attributes (Fig. 4), echoing the results of playback experiments that ant benevolent behaviors were induced significantly by different types of butterfly calls.

To assess the potential role of butterfly calls, we compared the calls of butterflies and ants, and performed the playback experiments. The discriminate analysis (Fig. 4) indicates that the pupal call and larval Type B calls of *S*. *lohita* adjoining with the ant call (part 1) form a group, while Type A and the ant call (part 1 + 2) overlap to some extent, implying that butterfly calls are similar to ant calls in call attributes. Playback of ant calls and all types of butterfly calls induce ant aggregation, further supporting that calls of both lycaenids and ants share a similar function and that the lycaenids are capable of producing similar calls of the ant possibly for the purpose of inducing the ant’s benevolent behavior^18,28,29^. Type A call of larvae and pupal call of *S*. *lohita* frequently occurred during our observation and invariably induced one of three ant’s benevolent behavioral reactions (antennation, aggregation, and guarding). This suggests that the two calls of *S*. *lohita* may serve as a primary call for *C*. *rogenhoferi*. Type B and Type C calls of *S*. *lohita*, however, occurred occasionally and only induce two types of ant responses (e.g., aggregation and guarding behavior), leading to a possibility of these calls likely functioning as communication cues with non-host recipients such as conspecific larvae or other co-inhabiting organisms^30^ in addition to communicating with the host ant. Despite variations in ant responses to the calls of the lycaenid, our results strongly indicate that vibrational cues play a significant role in the interactions between *S*. *lohita* and its attending ants.

It is unexpected that the playback of ant calls failed to induce strong behavioral reactions in ant workers. This may be explained by the fact that communication in ants typically involves both chemical and physical cues, and that vibrational calls alone may not be sufficient or intimate enough to produce a strong behavioral response. Equally unexpected is that no queen calls were recorded during the entire experiment, which prevents us from playing back queen’s calls. While it remains unclear whether a *C*. *rogenhoferi* queen can make calls, we did observe a functional stridulatory apparatus in queens that is structurally similar to that of workers (the only difference is the spacing between ridges, Table 2), implying that the *C*. *rogenhoferi* queen is certainly capable of producing vibrational signals. One explanation is that the environmental conditions while this study was being conducted may not be favorable in inducing calling behavior of the *C*. *rogenhoferi* queen. To our surprise, in playback experiments white noise was able to induce ant’s benevolent behavior. One of the possible explanations is that ants are generally rather sensitive to vibration, especially to unfamiliar vibrational signals^1,31,32^.

How *S. lohita* larvae make calls is still ambiguous thus far. Species in Riodinidae are known to produce signals using vibratory papillae, while species in Lycaenidae lack this structure^21^. It is believed that vibrational signals of lycaenid caterpillars may emanate from muscular contraction and air compression through the tracheae^33^. Our behavioral observation revealed that the larvae of *S*. *lohita* produced calls under any circumstances, and sometimes produced two types of call simultaneously. No apparent movements were observed in outer larval appearance while signals were being produced, thus leading us to speculate muscular contraction as a more likely signal-producing mechanism for *S*. *lohita*. Pupae of *S*. *lohita* produced calls from time to time in any condition while being recorded. Pupal calls are thought to have been generated from tooth-and-comb stridulatory organs between the fifth and sixth segments of abdomen in members of Riodinidae and Lycaenidae^34,35^. As no stridulatory movements of pupae while producing signals, future study should focus on the identification of the pupal vibrational organ and how pupal calls sustain high ant maintenance.

The stridulation apparatus of the ants represents the typical structural features as in other stridulating ants^36,37^. This consists of a file with transverse cuticular ridges on the anterior portion of the first gastral tergite, and a scraper that is formed by the posterior downward-bend posterior edge of the postpetiole^38^. Signal production is realized by moving the gaster up and down, which is achieved by the alternating action of two antagonistic muscle groups in the postpetiole: the paired dorsoventral muscles upon contraction pull the sternal part of the gaster forward and hence the tergal part backward, which results in one chirp. Contraction of the longitudinal muscle, on the other hand, pulls the tergal part of the gaster forward again and thus acts as the antagonist of the dorsoventral muscles^36–39^. Repeated stridulation will be the result of the alternating action of both muscles. These muscles represent the common muscular outfit that is known in the postpetiole of other ants, with the paired dorsoventral muscles corresponding with muscles 9 and 10, and the unpaired longitudinal muscle corresponding with muscle 8 in the study by Hashimoto^39^.

The finding of larvae of *S*. *lohita* producing three types of call is similar to what has been reported for *J*. *evagoras*^22^, although *J*. *evagoras* produce two types of pupal calls. However, the call attributes (both frequency and durations) differ in the two species. In contrast, *Maculinea rebeli*, as reported in Barbero, *et al*.^18^, produce only one type of larval call and one pupal call. Differences in call diversity and characteristics between *S*. *lohita* and other butterflies are in parallel with Riva, *et al*.^24^ that compared vibrational signals of 12 species from eight lycaenid genera (*Cacyreus*, *Lycaena*, *Cupido*, *Lycaeides*, *Scolitantides*, *Plebejus*, *Maculinea*, and *Polyommatus*) and concluded that the calls of Lycaenidae are species-specific, even for those of the same genus. Such differences may also hint that vibrational signals of caterpillars would serve as an applicable tool in species delimitation. Similarly, the temporal and spectral properties of vibrational signals of *C*. *rogenhoferi* differ from those of other ant species^18,40^. Ferreira, *et al*.^40^ reported that vibrational signals can be used in discovering cryptic species of ants as ants recognize conspecific signals and refuse heterospecific signals^41^. Combined with all empirical evidence, one may utilize vibrational pattern as a systematic character for taxa such as butterfly and ant. The practice of vibrational signals in α-taxonomy can also be seen in other insect taxa, such as treehoppers^42^, lacewings^43^, and psyllids^44^.

In Taiwan, there are two other myrmecophilous butterfly species that are closely related to *S*. *lohita*, namely *S*. *syama* and *S*. *kuyanianus*. Both species are believed to be obligate mutualists with their attendant ants, *C*. *popohana* and *C*. *amia* for the former, and *C*. *laboriosa* for the latter. This system provides a great opportunity to test interspecific competition for the vibrational niche between ants and butterflies. For example, whether these species make distinct and species-specific vibrational calls to attract their respective host ants remains unclear. Furthermore, one also can test if these call characters can be used for species identification.

## Methods

### Collection and lab rearing of butterfly and host ants

Ten to fifteen *S*. *lohita* females were collected from the montane areas in northern Taiwan during September 2014 and August 2015. All adult female butterflies were caged with the ant *C*. *rogenhoferi* and a branch of the host plant *Mallotus paniculatus* (Euphorbiaceae) in an 18.5 × 10.5 × 4.5 cm box for egg laying. Newly hatched larvae were separated individually to a branch of the host plant *M. paniculatus* in a 5.5 × 8 × 3 cm cage with daily provision of fresh *M. paniculatus* leaves and removal of frass.

The ant *C*. *rogenhoferi* builds a ball-shaped nest on various tree species in the montane areas of Taiwan. Twelve ant colonies were collected from the same areas where *S*. *lohita* were collected between July 2013 and October 2015. Every collected nest was searched in a detailed manner for the presence of the queen. Once found, the queen and the remainder of the colony were transferred and maintained in an artificial harborage made of plaster which is placed in a box (34 × 24 × 15 cm). Fluon was carefully applied onto the inner walls of the box to prevent the ants from escaping. All ant colonies were maintained at 25 °C with a schedule of 12-hour light and 12-hour dark with daily supplement of water, honey solution and mealworms. All ant colonies were allowed at least one-month acclimatization period.

### Signal recording experiment

The 22 6^th^ instar larvae and 16 pupae were taken to a noiseless recording studio for signal recording. The larvae and pupae were placed on a branch of their host plant. The 3 M Scotch mounting putty was used to secure the branch (ca. 15 cm height) to reduce its movement. After 5-min acclimatization, the recording started and lasted 20 mins. The signal recording method followed Liao and Yang^45^ and Liao, *et al*.^46^. Vibrational signals were recorded using a gramophone stylus through an amplifier (Lzban, DRA-455, China) and the stylus slightly touched the surface of the host plant leaf. The signals were saved in a dictaphone (Laxon, USB-F20, Taiwan). The number of signal-producing individuals was denoted as *N* and the number of signal used in sequential analysis was denoted as Σ in the Results section. For the recording of ant workers, the six entire artificial ant harborages were taken to the noiseless recording studio for the signal recording. A branch of *M. paniculatus* (ca. 15 cm) was put in the nest and one mealworm was positioned on the leaf to stimulate ant calls. After 30-min acclimatization, the recording started and lasted 30 mins. The gramophone stylus was directly put on the plant. For the case of ants, *N* was representative of the number of artificial ant harborages with signal production. Three ant queens were tested with the recording procedure of caterpillar as described earlier. All the resulting calls were processed through a two-stage noise reduction before analysis. Sampling rate for signal recording was 48,000 Hz and bit depth was 32-bit resolution. Recordings can be accessed in the Supplementary Audios.

### Playback experiments

The behavioral experiment setup followed Barbero, *et al*.^18^, and was carried out in a 10 × 10 × 8 cm acrylic arena. A loudspeaker from which the speaker cone has been removed was attached through a hole in the side wall, and sealed on the outside with the scotch removable mounting putty. Ten workers from the same colony were placed in the arena and allowed to settle for 10 minutes before the test calls were played from a MP3 player. The calls were composed of loops of the original recordings, with the volume adjusted to the level reached during the recording.

Three types of larval call, pupal call and ant call were played to the ants. A white noise which was the background sound generated by the recording machine was used as control, a procedure to test if ants react to a potentially meaningless signal. A silence treatment was added to the experiment as a second control and defined as intercept in regression analysis. Ants’ behavioral reactions were recorded and categorized as attack, antennation (antenna drumming and vibrating), aggregation (moving toward the speaker) and guarding (standing by the speaker and lifting the gaster). The behavioral experiment lasted for 10 mins, during which different ants’ behavior reactions toward the speaker were recorded every minute. The number of each reaction of every minute was summed up to represent the level of each behavior during the 10-minute period (*N* = 10 colonies, each colony was tested for 3 times, with a total of 30 trails for each treatment).

### Statistical analysis and plotting

Dominant frequency, pulse duration and pulse period of all vibrational signals from pupae, larvae and ants were measured by Audacity 2.1.0 to obtain call characteristics. The dominant frequency is the strongest frequency in the spectrogram. The pulse duration is the time length of pulse or pulse trains specific for Type A call of caterpillar, while the pulse period is the time length between the starting points of two consecutive pulses. Call characteristics of both caterpillars and ants were analyzed by using a Mann-Whitney *U* test and Kruskal-Wallis tests by Past 3.14^47^. A *P* value < 0.05 was considered significant. Discriminant analysis (DA) was performed using Past 3.14 to reveal signal characteristics of different types of call. A multivariate normality test was conducted, and the raw data did not match the normal distribution (Mardia’s test, statistic = 484.1, df = 10, *P < *0.001). Hence, all data were transformed (log(n + 1)) prior to the DA^48^. To determine whether an ant was attracted by caterpillar signals, the association between ant behavior and playback signal was analyzed using multiple linear regression analysis using Past 3.14. Figures were produced using Sigma Plot 10.0 (Systat Software, San Jose, CA). The oscillogram and spectrogram of call signals were generated through the Matlab 8.0. (R2012b, The MathWorks, Natick, MA, USA). For audio file reading we used scripts by Ellis^49^. The script for noise reduction and plotting was modified from Vincent^50^ and Zhivomirov^51^. Spectrograms were computed using Fast-Fourier transformation (FFT) with a 1024-point window size (Hann window) and a 97% overlap.

### Histological examination

From one alate gyne, one dealate queen and four workers, the middle part of the body was separated by making a transverse cut at the level of the midlegs anteriorly and behind the first gastral segment posteriorly. The tissues were fixed in cold 2% glutaraldehyde, buffered at pH 7.3 with 50 mM Na-cacodylate and 150 mM saccharose. After postfixation in 2% osmium tetroxide in the same buffer, tissues were dehydrated in a graded acetone series and embedded in Araldite. Serial longitudinal semithin sections with a thickness of 1 μm were made with a Leica EM UC6 microtome and stained with methylene blue and thionin. The sections were observed and photographed with an Olympus BX-51 microscope. For each individual, we measured the length of the stridulation file and distance between ridges and counted the number of ridges.

## Supplementary information

Larval type A call

Larval type B call

Larval type C call

Pupal call

Ant call

The associations between three behavioral responses of the ant Crematogaster rogenhoferi and different playback signals were analyzed by using multiple regression analysis
