# Supplementary material for: Vibrational communication between a myrmecophilous butterfly Spindasis lohita (Lepidoptera: Lycaenidae) and its host ant Crematogaster rogenhoferi (Hymenoptera: Formicidae)
Source: Sci Rep. 2019 Dec 6;9:18548. doi: 10.1038/s41598-019-54966-6 (PMC6897957; doi:10.1038/s41598-019-54966-6)
Supplement: Supplementary file 6 — The associations between three behavioral responses of the ant Crematogaster rogenhoferi and different playback signals were analyzed by using multiple regression analysis [file 41598_2019_54966_MOESM6_ESM.docx]

**Vibrational communication between a myrmecophilous butterfly *Spindasis lohita* (Lepidoptera: Lycaenidae) and its host ant *Crematogaster rogenhoferi* (Hymenoptera: Formicidae)**

**Yueh-Hsien Lin^1,+^, Yi-Chang Liao^2,+^, Chin-Cheng Scotty Yang^3^, Johan Billen^4^, Man-Miao Yang^2^, Yu-Feng Hsu^1,*^**

^1^College of Life Science, National Taiwan Normal University, 162 Hepingtung Rd., Taipei, 106 Taiwan

^2^Department of Entomology, National Chung Hsing University, 145 Xinda Rd., Taichung 402, Taiwan

^3^Research Institute for Sustainable Humanosphere, Kyoto University, Gokasho, Uji, Kyoto 611-0011, Japan

^4^KU Leuven, Zoological Institute, Naamsestraat 59, box 2466, B-3000 Leuven, Belgium

^*^Corresponding author email: frankhsu520406@gmail.com

^+^These authors contributed equally to this workTable S1. The associations between three behavioral responses of the ant *Crematogaster rogenhoferi* and different playback signals were analyzed by using multiple regression analysis

| Ant response |  | Coefficient | t | P value |
| --- | --- | --- | --- | --- |
| Antennation | Type A call | 0.97 | 2.63 | < 0..01 |
|  | Type B call | 0.47 | 1.27 | 0.205 |
|  | Type C call | 0.07 | 0.18 | 0.856 |
|  | Pupal call | 2.07 | 5.63 | < 0.001 |
|  | Ant call | -0.40 | -1.09 | 0.277 |
|  | White noise | 0.53 | 1.45 | 0.148 |
| Aggregation | Type A call | 10.73 | 8.27 | < 0.001 |
|  | Type B call | 13.73 | 10.58 | < 0.001 |
|  | Type C call | 13.53 | 10.42 | < 0.001 |
|  | Pupal call | 11.53 | 8.88 | < 0.001 |
|  | Ant call | 6.07 | 4.67 | < 0.001 |
|  | White noise | 4.80 | 3.70 | < 0.001 |
| Guarding | Type A call | 4.70 | 2.24 | < 0.05 |
|  | Type B call | 11.00 | 5.23 | < 0.001 |
|  | Type C call | 9.67 | 4.60 | < 0.001 |
|  | Pupal call | 7.00 | 3.33 | < 0.01 |
|  | Ant call | -0.20 | -0.10 | 0.924 |
|  | White noise | 6.80 | 3.23 | < 0.01 |
